# An Automatic Classification Method on Chronic Venous Insufficiency Images

**DOI:** 10.1038/s41598-018-36284-5

**Published:** 2018-12-18

**Authors:** Qiang Shi, Weiya Chen, Ye Pan, Shan Yin, Yan Fu, Jiacai Mei, Zhidong Xue

**Affiliations:** 1 School of Software Engineering, Huazhong University of Science and Technology, Wuhan, 430074 China; 2 Vascular surgery of Shanghai Sixth People’s Hospital affiliated to Shanghai Jiao Tong University, Shanghai, 200233 China; 3 School of Mechanical Science and Technology, Huazhong University of Science and Technology, Wuhan, 430074 China

## Abstract

Chronic venous insufficiency (CVI) affect a large population, and it cannot heal without doctors’ interventions. However, many patients do not get the medical advisory service in time. At the same time, the doctors also need an assistant tool to classify the patients according to the severity level of CVI. We propose an automatic classification method, named CVI-classifier to help doctors and patients. In this approach, first, low-level image features are mapped into middle-level semantic features by a concept classifier, and a multi-scale semantic model is constructed to form the image representation with rich semantics. Second, a scene classifier is trained using an optimized feature subset calculated by the high-order dependency based feature selection approach, and is used to estimate CVI’s severity. At last, classification accuracy, kappa coefficient, F1-score are used to evaluate classification performance. Experiments on the CVI images from 217 patients’ medical records demonstrated superior performance and efficiency for CVI-classifier, with classification accuracy up to 90.92%, kappa coefficient of 0.8735 and F1score of 0.9006. This method also outperformed doctors’ diagnosis (doctors rely solely on images to make judgments) with accuracy, kappa and F1-score improved by 9.11%, 0.1250 and 0.0955 respectively.

## Introduction

Chronic venous insufficiency (CVI) affect 15–25% of the general population and occur significant financial burdens on the healthcare system, costing up to a billion dollars each year in the United States^1^, and often occur in the lower limbs. Clinical manifestations of lower limbs CVI include superficial femoral vein dilatation or varicose veins, leg weakness, heavy, tenderness, edema, skin nutritional changes, venous ulcers^2^. In addition, CVI can involve the superficial vein, traffic vein, and deep vein or even the entire lower limb venous system. This disease can be caused by primary or secondary factors, and venous reflux or proximal obstruction is the main hemodynamic change^3^. Due to the diversity of clinical manifestations and the complexity of pathophysiological changes, correct diagnosis is the foundation to provide accurate treatment^4^.

The commonly used classification system of CVI includes the CEAP (Clinic, Etiologic, Anatomic and Pathophysiological) system and VCSS (Venous Clinical Severity Score) scoring system^5,6^. The levels in clinical manifestations in CEAP system range from C0 (symptoms, no signs) to C6 (active ulcers). The CEAP system has been accepted widely and been applied in clinical diagnosis, and effectiveness evaluations. However, the CEAP classification is descriptive, and does not register objective complaints and is not a severity score^7^. Thus, Rutherford improved the CEAP system and proposed the VCSS system which includes 10 scoring items (pain, edema, venous claudication, pigmentation, scleroderma, ulcers, ulcers diameter, duration of disease, recurrence and quantity)^8^ with each item ranging from 0 to 3. The higher the total score, the more serious the CVI’s severity. However, VCSS is more comprehensive and more responsive to venous disease. These classification systems are designed to offer doctors indicative information when they make decisions. However, the application of these scoring systems is depend on doctors’ experience and subjective judgement. Furthermore, these scoring systems require professional knowledge, which is difficult for patients to apply self-evaluation.

We propose an automatic classification approach based on optical images to evaluate CVI’s severity, named CVI-classifier. In CVI-classifier, a multi-scale semantic model^9^ is used to explore the middle semantic information contained in low-level features such as texture, shape, color and spatial relationship. A scene classifier based on middle-level semantic features is adopted to estimate CVI’s severity as high-level semantics. In order to improve classification accuracy, feature selection method based on high-order dependency is introduced to obtain the optimal feature subset. This automatic classification approach provide patients with an easy access to online medical services and doctors can work out appropriate treatment from large amount of similar medical records.

## Related Work

With the rapid development of modern medical imaging technology, automatic medical image classification has become more and more important for diseases diagnosis, medical references and surgical planning^10–12^. Medical image classification approaches have already been used for cancer detection^13^, stroke identification^14^, Alzheimer’s disease^15^, etc., however, no classification approach for CVI images exists at present. Moreover, traditional medical image classification methods are mostly based on low-level image features, such as color, texture and shape^16^. These low-level features cannot reflect certain hidden information in the medical images, creating the “semantic gap” problem between low-level features and high-level information, which is one of the biggest challenges for medical image classification^17^.

To reduce the semantic gap, Bag of Visual Word (BoVW) model^18,19^ is introduced to form middle-level features for describing high-level semantics. A typical procedure of BoVW model is illustrated as follows:

1. an image is sampled efficiently with various local interest point detectors^20^ or dense regions^21^, and is described by local descriptors^22^;
2. A codebook consisting of several codewords is learned with clustering techniques, such as K-means and spectral clustering, to quantize the local features into discrete values;
3. The visual histogram achieved by calculating the occurring frequency of each visual word is used to represent the medical image.

BoVW has been used in medical image classification tasks and achieved inspiring performance. However, there are still some design choices to make in different steps of BoVW model, including the choice of local feature descriptor, dictionary learning and middle-level semantic image representation.

The delicate design of local features depicting different aspects of visual appearance is the basis for the success of BoVW models in medical image classification. Due to its invariance to translation, illumination, and scale, SIFT and its improved versions, such as SURF, become the most popular local descriptor^23,24^. Considering the dimension of SIFT-based local features is high, affine moment invariants that can produce small-sized compact feature vectors with minimum information redundancy are proposed^25^. The above descriptors are computed based on the local region around some key points, however, most medical images have little meaningful key points and structures in the lesions, so patch-based BoVW models are proposed to medical image classification tasks^26^. In these approaches, medical images are partitioned into multiple blocks and local descriptors are calculated according to block intensity. All these types of features have been proven to be very powerful descriptors to detect and describe local features in images. However, the single local descriptor may perform poorly when the image contains complex background due to the fact that a portion of extracted features may come from the noisy background. In practice, a combination of multiple local features^27^ often yields better performance on image representation.

Dictionary learning is another a critical component of BoVW, and can be divided into unsupervised and supervised approaches^28–30^. Unsupervised clustering techniques, such as the K-means, K-median clustering, mean-shift clustering, hierarchical K-means, agglomerative clustering^31–34^, are usually used for constructing the visual dictionary. In these approaches, the feature vectors are clustered and the centroids of the clusters are used to form the codebook. One of the common features of these unsupervised methods is that they only optimize an objective function fitting to the data but ignoring their class information. Therefore, this reduces the discriminative power of the resulting visual dictionaries. To create more discriminative visual words, supervised dictionary learning techniques that optimize the dictionary for a specific classification problem are proposed, and are proved to outperform corresponding unsupervised methods^35^. In recent work, Saghafi^36^ proposed a concept space to illustrate the semantic relations between the visual codewords. They apply generative models such as latent semantic analysis (LSA) and probabilistic latent semantic analysis (pLSA) to discover the latent semantic relations between the initial codewords. N. Passalis^37^ generalized and formulated the BoVW model as a neural network, which is composed of a Radial Basis Function (RBF) layer and an accumulation layer. Moreover, the proposed model can be trained in the supervised fashion when it is followed by a multilayer perceptron (MLP) as classification layer. B. Fernando^38^ introduced the Gaussian mixture model for codebook generation that not only generalizes the K-means algorithm by allowing soft assignments, but also exploits supervised information to improve the discriminative power of the clusters. R. Ji^39^ proposed a task-dependent codebook compression framework to reduce dimension of BOVW, based on the supervise labels coming from the classification task. Although supervised learning has been introduced to codebook generation, local labels are still ignored, which impedes its overall performance.

After learning the visual words, all the encoded local features will be pooled to form an image-level feature vector. However, a global histogram only reflects the holistic distribution of codewords, the information about the spatial layout is lost, which could be important cues for image classification. To take advantage of spatial information, Lazebnik *et al*.^40^ proposed a spatial pyramid matching (SPM) framework by partitioning the image into increasingly fine sub-regions and computing histograms of local features found inside each sub-region, which has become a widely used strategy to incorporate spatial information. To further improve the SPM method, Zhou *et al*.^41^ incorporated a multiresolution representation into the traditional BoVW model by constructing multiple resolution images and represented each resolution image by features extracted from the sub-regions of two modalities of horizontal and vertical partitions. Although the above methods fully consider the spatial characteristics of the image, they do not include local semantic that can effectively eliminate the ambiguity of the local features. In order to further exploit local concepts of images, Y. Tanaka^42^ proposed a multi-level resolution semantic modeling method for automatic scene recognition, which constructs global image representation with concept probabilities of local regions in each level resolution, and combine semantic representations of multi-level resolution for scene recognition. Although these variants of the original BoVW model have a good descriptive ability to depict spatial layout and semantic information of images, they are designed mainly for natural image scene classification, and seldom have they been applied in the field of medical image classification.

To this end, considering local lesions of CVI images have meaningful semantics and several sizes, this paper proposes a framework for discovering multi-level local semantics to improve the performance of automatic CVI image classification. Differences from traditional BoVW models include: 1) Various features of local region are combined to better exploit discriminative appearances. 2) Supervised learning approach directly based on local-regions’ labels is used to generate visual vocabulary. 3) The final representation of global image is modeled by the combination of middle-level semantics that are calculated by counting frequencies of local concepts at each level resolution.

## Results

In our experiments, all 221 images come from 217 patients’ medical records that were collected by three professional doctors of vascular surgery of Shanghai Sixth People’s Hospital affiliated to Shanghai Jiao Tong University. Among the 217 patients, four of them are provided with two images taken from different perspectives. Therefore, the number of images is not equal to the number of patients. These leg images in medical records are taken by these professional doctors. In order to ensure the accurate recording of the patient’s situation, some photographing conditions are required, including sufficient lighting, simple background, and full coverage of leg area. Since these images vary in size and have different backgrounds and lightness, they are resized to the same size (250 × 700) according to the ratio of leg’s length and width, and are preprocessed including background reduction and lightness normalization.

In order to train and evaluate concept classifier, the preprocessed images are divided into patches with size 15 × 15, 25 × 25 and 50 × 50 respectively, and these patches are annotated as described in *method* section. Furthermore, the ground truth labels for global images come from the medical records that are not only determined by the image data, but also other evidence, e.g. color ultrasonography of deep venous at lower extremities, venography of lower extremity etc. The labels of these images are classified ‘mild’, ‘moderate’ and ‘severe’ categories according to the following treatments for patients. The patients in mild situation do not need to handle, or wear elastic stockings, the ones in moderate situation need simple surgery, and the ones in severe situation need a complex, systematic treatment plan. The abovementioned dataset includes 59 images labeled as mild situation, 97 images denoted as moderate class, and 65 images belonging to severe situation. The image dataset and relevance labels can be downloaded from http://isyslab.info/CVI/CVI-img-datasets.zip and https://github.com/shiqiangdqq/CVI-classifier/tree/master/CVI-imgdatasets.

Classification accuracy, Kappa coefficient and F1-score are used to evaluate the corresponding classifiers. Suppose *M* to be a confusion matrix, *r* is the number of rows, and *x*_*ii*_ is the value of row *i* and column *i*, *x*_*i*+_ means the sum value of the row *i*, and *x*_+*i*_ means the summary value of column *i*, *N* is the number of total samples, then, the classification accuracy (Eq. 1) and Kappa coefficient (Eq. 2) are calculated as follows:

1 $$acc=\frac{\sum _{i=1}^{r}{x}_{ii}}{N}$$

2 $$kappa=\frac{N\sum _{i=1}^{r}{x}_{ii}-\sum {x}_{i+}{x}_{+i}}{{N}^{2}-\sum {x}_{i+}{x}_{+i}}$$

Since the problem in this paper is multi-class classification, we use the average F1-score to evaluate classifier’s performance^43^. Suppose *C* = {*C*_1_, *C*_2_, ..., *C*_|*M*|_} is the category set, and |*M*| is the category number. For a binary-classification by treating *C*_*j*_ as positive class and the other categories as negative class, the precision ${P}_{j}=\frac{TP}{TP+FP}$ and recall ${R}_{j}=\frac{TP}{TP+FN}$ are calculated respectively where *TP*, *FP*, *FN* denote true positive, false positive and false negative, and then, the F1-score ${F}_{1}^{j}=\frac{2{P}_{j}{R}_{j}}{{P}_{j}+{R}_{j}}$ is computed. In addition, for the evaluating whole performance of all classes, the average F1-score (Eq. 3) is calculated as follows:

3 $${F}_{1}^{avg}=\frac{\sum _{j=1}^{|M|}{F}_{1}^{j}}{|M|}=\frac{\sum _{j=1}^{|M|}(2{P}_{j}{R}_{j}/({P}_{j}+{R}_{j}))}{|M|}$$

In this paper, all the experiments were conducted on MATLAB 2017b environment running on a computer with Intel Core i7 3.4 GHz processor and 32 GB memory.

### The Performance of Concept Classifiers

**Figure 1:**
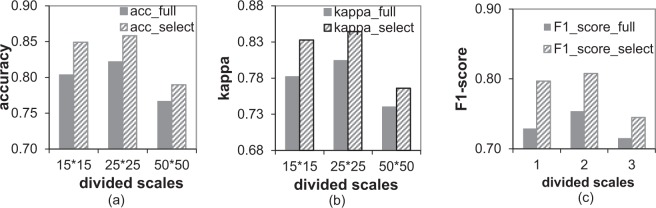
Performance comparisons of the two concept classifiers at different divided scale. (**a**) Accuracy comparisons of concept classifiers based on all features and ones based on selected features at each divided scale; (**b**) kappa coefficient comparisons of concept classifiers based on all features and ones based on selected features. (**c**) F1-score comparisons of concept classifiers based on all features and ones based on selected features at each divided scale.

**Figure 2:**
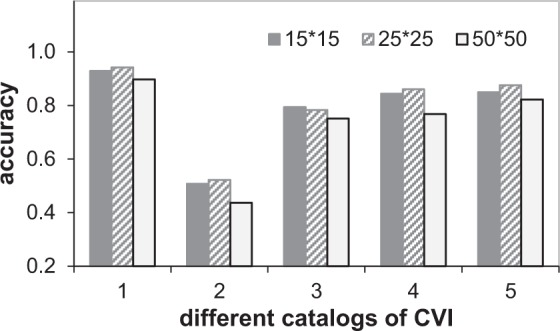
The comparison of concepts accuracies at each divided scale. 1 - normal skin, 2 - reticular vein or telangiectasia, 3 - varicose veins, 4 - pigmentation or edema and 5 - vein ulcers.

In order to train and test concept classifier, 2/3 samples of the annotated patches which were labeled by three professional doctors are randomly selected for training, and the remaining 1/3 samples are used for testing. In addition, the above process is repeated 20 times, and the averages of the classification accuracy, kappa coefficient and F1-score are used to evaluate the classification performance of concept classifier. After prior comparisons of multiple kernel functions (Gaussian kernel functions with different parameters and different-order polynomial kernel functions) and two multi-class classification strategies (one-vs-one and one-vs-all), we chose a quadratic polynomial kernel as the SVM classifier kernel function, and adopted one-vs-one strategy to achieve multi-classes classification. For the comparison, the *CC_all* which means the concept classifier is trained using all region features in each divided scale, and the *CC_selected* which means the concept classifier is trained based on the optimized subset which is selected from all region features by the approach proposed in the *method* section. The accuracy averages and kappa averages of 20 repeated trains and tests are shown in Figs 1 and 2, respectively.

From the results, we can find as follows. 1) For each scale, the classification accuracy and Kappa coefficient of *CC_selected* are higher than the ones of *CC_all*, since the selected feature subset does not contain redundant and noisy features, the classification accuracy and generalization of the concept classifier trained with these features are improved. 2) For each concept, the highest classification accuracy of varicose veins is at divided scale 15 × 15, the best accuracy of reticular vein or telangiectasia is at divided scale 25 × 25, the accuracies of vein ulcers and pigmentation at divided scale 25 × 25 and 50 × 50 are much better than the ones at divided scale 15 × 15. These results can prove that the proper size of the image patch is important for classifying specific CVI’s category. 3) The accuracy of normal skin at all scales are more than 90%, because the visual characteristics of normal skin are different from others. Therefore, CVI-classifier uses multiple concept classifier based on optimized features to yield the middle-level semantics.

### The Performance of Scene Classifier

In our experiments, we choose the concept classifier with best accuracy in the above 20 repeated trainings to classify all image patches for image representation. Since our dataset is small, we want to use as many samples as possible to explore meaningful patterns in scene classifier training. In order to guarantee the scene classifier’s generalization, after classifying the patches of all images using the selected concept classifier, we use 2/3 samples of our image dataset as training set and the remaining 1/3 samples as testing set. Considering that the 2/3 patch samples are distributed in all images and the proportion of these samples is very small, the samples for training concept classifier is not as the same as for training scene classifier. The above scene classifier training is repeated 20 times, and the averages of accuracy, kappa and F1-score are utilized to evaluate the performance of scene classifier. After prior comparisons of multiple kernel functions (Gaussian kernel functions with different parameters and different-order polynomial kernel functions) and two multi-class classification strategies (one-vs-one and one-vs-all), we chose a linear kernel as the SVM classifier kernel function, and adopt one-vs-one strategy to achieve multi-classes classification. As the category of image patch is related to its position, we count the concept occurrence frequency in the upper and lower part of the CVI image respectively as represented in Methods section. Finally, the global image representation is achieved by combining the region-wise image representation of each divided scale. The dimension of the new image representation is 45, including the COVs from whole image at all scales (3 × 5 = 15), and the COVs from the lower and upper image at all scales (3 × 10 = 30). Because of the training strategy mentioned above, the feature selection process is carried out for each repeated experiment. The comparison of the average accuracy and kappa coefficient of 20 times is displayed in Table 1. The confusion matrixes of 20 times are shown in Tables 2 and 3. The average number of selected features is 32, which is smaller than the dimension of original feature sets.

**Table 1:** The Performance of Scene Classifier Based on Selected Features From Combined COVs.

	Accuracy	Kappa	F1-core	Number of Features
Full Features	88.82%	0.8446	0.8773	45
Selected Features	90.92%	0.8735	0.9006	32

**Table 2:** Confution Matrix Based On All Semantic Features For Scene Classifier.

		Classification in %		
		mild	moderate	severe
true class	mild	92.14	7.38	0.48
	moderate	8.16	81.84	10.00
	severe	1.66	7.78	90.56

**Table 3:** Confution Matrix Based On Optimized Features For Scene Classifier.

		Classification in %		
		mild	moderate	severe
true class	mild	94.05	5.24	0.71
	moderate	6.58	84.74	8.68
	severe	1.25	6.39	92.36

From the above results, we can conclude as follows. 1) the average accuracy is 90.92%, the average kappa coefficient is 0.8735, and the average F1-score is 0.9006 for determining the severity of CVI using the optimized feature subset. 2) For mild, moderate and severe category, the classification accuracy is increased by 1.91%, 2.90%, and 1.80% respectively when scene classifier uses optimized features. 3) The average number of selected features for 20 experiments is 32, which is smaller than the dimension of all features. Since the redundant and noisy features are removed, the optimized feature subset leads to the better performance. In CVI-classifier, the classification performance is improved by feature selection approach.

In order to verify the efficiency of CVI-classifier, we used the trained concept classifier and scene classifier to classify the 50 randomly selected images. The average times of image representation calculating, scene classification at each divided scale are counted. The times of achieving middle-level image representations are 5.4493 seconds for 15 × 15 scale, 5.6567 seconds for 25 × 25 scale, and 5.0736 seconds for 50 × 50 scale. Moreover, the average time of classifying images is 0.0081 seconds. In addition, we use MATLAB Parallel Computing Toolbox^44^ to achieve multi-scale middle-level image representation. This parallel computation needs 5.802 seconds. That means if we use parallel computing to extract multi-scale image representation features on the server, the total computation time will be reduced by almost 2/3, which can satisfy the needs of mobile devices.

### The Comparison with Other Method

In this subsection, CVI-classifier is compared with the semantic modeling proposed in^45^ and the K-means clustering based multi-resolution bag-of-features model in^41^ to prove its better performance.

The semantic modeling in^45^ uses the grid with a fix size to divide image, while in CVI-classifier, the grids with several sizes are used to divide image. According to the basic idea of the semantic modeling, in Table 4, the scene classification results at each divided scale and the combination of all divided scales are reported. For fair comparison, the same feature selection approach is adopted in all experiments.

**Table 4:** The Comparisons Of Classification Performance At Each divided Scale and The Combination Of All Divided Scales.

Symbol	Accuracy	Kappa	F1-score
15*15	84.93%	0.7890	0.8288
25*25	83.88%	0.7747	0.8216
50*50	87.04%	0.8172	0.8542
Combined	90.92%	0.8735	0.9006

From the above results, we can conclude as follows. 1) the classification results at each divided scale are different, because the middle-level semantic features depend on the divided scale. 2)Although the best accuracy and kappa coefficient are 87.04% and 0.8172 at single divided scale, these results are lower than the ones based on the combination of all divided scales, since the combined middle-level features are more distinguishable, and can provide meaningful and useful information for scene classification.

**Figure 3:**
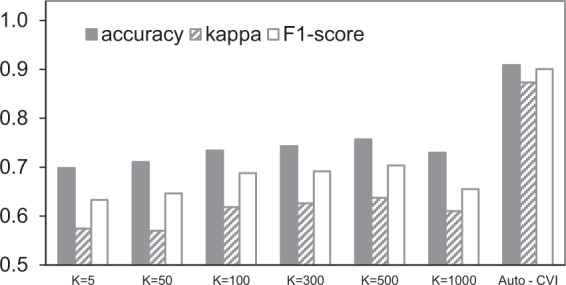
The comparisons of classification performance of CVI-classifier and the K-means based approach (k = 5, 10, 50, 100, 300, 500, 1000).

In comparison with the method proposed by L. Zhou *et al*.^41^, we replace the concept classifier with K-means clustering method in CVI-classifier to generate the visual codebook. The numbers of cluster center are 5, 10, 50, 100, 200, 300, 500 and 1000 respectively, and the classification result is shown in Fig. 3. The best result of the K-means based method is achieved with the cluster number of 500, and the accuracy, kappa and F1-score are 75.68%, 0.6376 and 0.7039 respectively. While the SVM-based method achieved 90.92%, 0.8735 and 0.9006 respectively, and they are 15.24%, 0.2359 and 0.1967 higher than the best result from K-means-based method. The reason for this is that these local semantic annotations produced by supervised learning methods are more distinguishable for deciding the severity of CVI than the ones based on unsupervised learning approaches such as K-means clustering.

### The Comparison with Doctors’ Judgments

**Figure 4:**
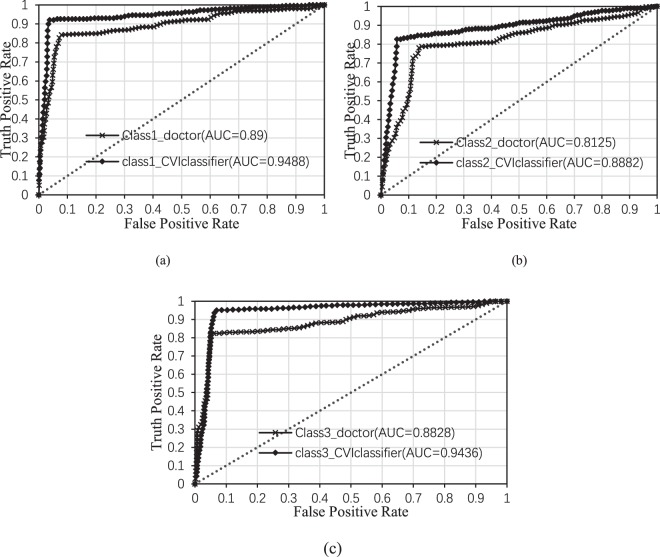
he ROC curves for all categories by doctors and CVI classifier.

In order to validate our method, the results are compared with the judgements of a group consisting of 20 doctors who have prior knowledge about CVI (but not the images in our dataset). They have long been engaged in microcirculation clinical surgeons and belong to the group of varicose vein, Chinese Society of microcirculation. These 20 doctors made their choices only based on optical images, not including evidences from other diagnostic methods such as color ultrasonography of deep venous at lower extremities and venography of lower extremity. In the process of evaluation, there is no communication between the other 20 doctors and the 3 doctors who participated in image annotation, there is also no communication among 20 doctors In our experiments, the CVI images are randomly sorted, and each doctor provides the diagnostic results for CVI images independently. The average classification accuracy of the human group is calculated to evaluate their performance. The single category and overall classification accuracy of each approach are shown in Table 5. In addition, as shown in Fig. 4, we plot the ROC curve and compute its AUC (Area Under Curve) for each category for comparing the performances of 20 doctors and CVI-Classifier.

**Table 5:** The Comparisons Of Classification Performance At Each divided Scale and The Combination Of All Divided Scales.

	mild	moderate	severe	overall	F1-score_avg	kappa
doctors	84.24%	78.68%	82.14%	81.81%	0.8051	0.7485
CVI-classifier	94.05%	84.74%	92.36%	90.92%	0.9006	0.8735

The accuracies of CVI-classifier and doctors are 90.92% and 81.81% respectively. Since CVI-classifier is based on quantitative indicators computed from objective image, the accuracies of CVI-classifier for mild, moderate and severe category are 94.05%, 84.74% and 92.36% respectively, which are 9.81%, 6.06% and 10.22% higher than the conclusions drawn by doctors. The classification performance of doctors is less than 100%, and these diagnoses are disagreement with actual ground truth labels, as these 20 doctors, which have different personal experiments and prior knowledge, only utilized optical images for diagnosis. However, compared with 20 doctors’ performance, CVI-classifier achieved a higher classification results due to the elimination of some subjective factors. To further analyze the performance of the classifier, we also compared the average of F1-score and the AUC for each class. The average F1-score of CVI-classifier is 0.9006, and is improved by 0.0955 compared with 20 doctors. For each categories, the AUCs are 0.9488 for mild, 0.8882 for moderate and 0.9436 for severe. Compared with 20 doctors, the AUCs are improved by 0.0588 for mild, 0.0757 for moderate and 0.0608 for severe respectively. These indicators show that CVI’s classification performance is superior.

**Figure 5:**
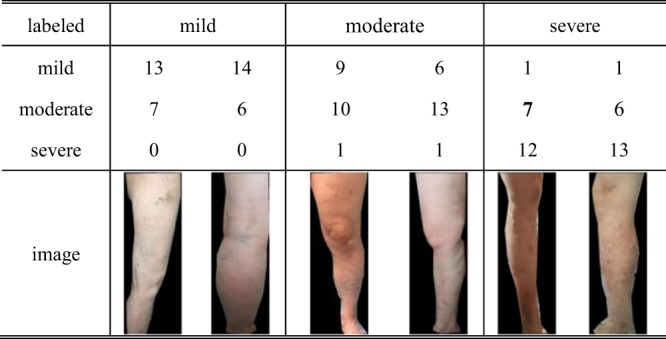
The number of doctors for predicting each severity category on some images.

To further illustrate the impact of physician subjective factors on classification, we present the confusion matrix of 20 doctors’ classification and some examples of misclassified images in Table 6. From this Table, we can see that 12.20% mild images are classified as moderate class, 14.92% moderate images are predicted as mild class, and 14.86% severe images are annotated as moderate class. Because each doctor have different comprehensions for ‘reticular vein or telangiectasia’ and ‘varicose veins’, these two classes are misclassified by several doctors. For example in Fig. 5, the mild image in column one is classified as moderate class by 7 doctors, and the moderate image in column three is annotated as mild class by 9 doctors. Since changed colors, closed and active ulcers included in skin changes are often affected by conditions when the images are taken, doctors may not notice small ulcers or distinguish the changed color from the darker skin. For instance, the serious images in column 5 and 6 are annotated as heavy class by 7 and 6 doctors, respectively. To further analyze these conflicts, we used Cohen’s kappa coefficients to calculate the inter-rater reliability between each doctor and the ground truth^46^. In addition, we also used the same approach to calculate the inter-rater reliability between the ground truth and the classification results of CVI-classifier. The average, maximum and minimum value of Cohen’s kappa coefficients for 20 doctors are 0.7232, 0.9151 and 0.3385 respectively. While the Cohen’s kappa coefficients between the ground truth and the classification results of CVI-classifier is 0.8580. Although this value is lower than doctors’ maximum, it is higher than doctors’ average. This comparison indicates that the performance of the classifier is within the variability of manual experts. Therefore, in order to reduce the above conflicts, CVI-classifier use computational and quantitative ways to find out the difference between several classes.

**Table 6:** Confution Matrix Of 20 Doctors’ Classifiction Result For CVI Dataset.

	Classification in %			
		mild	moderate	severe
true class	mild	84.24	12.20	3.56
	moderate	14.92	78.68	6.40
	severe	3.00	14.86	82.14
	precision:	**81.01**	**66.30**	**93.75**

## Discussion

In this study, we proposed an automatic classification approach for classifying CVI images to provide information on CVI’s severity. This automatic classification approach is based on multi-scale sematic modeling, in which the low-level image region features at several divided scales are mapped to middle-level semantic features which are used to predict CVI’s severity. In order to improve the classification performance, the high-order dependency based feature selection approach is introduced in training the concept and scene classifiers. Experimental results show that CVI-classifier outperforms other methods, such as single semantic modeling, K-means clustering based multi-resolution bag of features model, and even doctors’ judgments (solely based on images), in terms of accuracy, kappa coefficient, F1-score, etc.

The advantages of CVI-classifier are objective and low-cost. It is not like the Villalta^47^ scoring system that rely on doctors’ assessments for various symptoms. CVI-classifier make decisions only based on objective information. Compared with professional equipment such as ultrasonography, taking photos by mobile phone, pad and camera is low-cost. Furthermore, the requirements for taking photos including simple background, uniform illumination, and covering entire leg are easy to satisfy. In addition, CVI-classifier can automatically adjust the brightness and shallow of the photos. This is important for large-scale patients to use it.

CVI classifier is useful for patients and doctors. Patients with basic medical knowledge can get the medical advisory service from compute system. This can help patients to seek medical care in time. For doctors, the backend server automatically recommends the patients to doctors according to the severity though analyzing the uploading images by the patients in the big data era.

In the future, we will accelerate our approach using GPU-based parallel algorithm and introduce deep features to the framework of CVI-classifier. We will also construct a large-scale CVI image database, and mine more information from images such as 3D information to achieved better classification. In addition, considering the importance of subjective assessment we will develop a fusion system that will combine objective and subjective aspects for CVI classification.

## Methods

**Figure 6:**
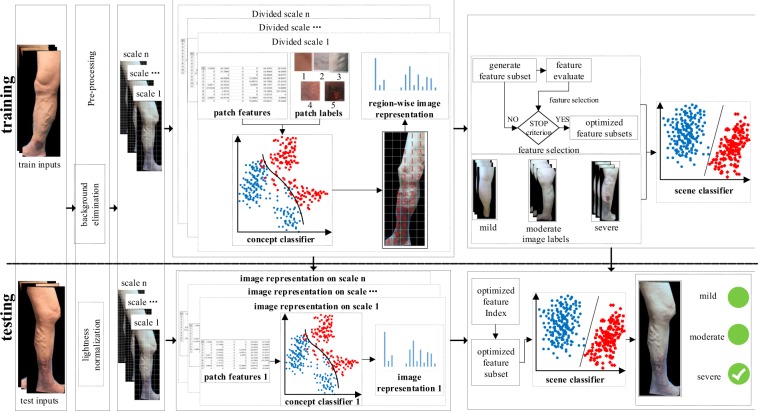
The flowchart of CVI-classifier. The concept classifier is trained to map low-level image region features to middle level semantic features, and the scene classifier is trained based on semantic features to achieve high-level interpretations of the image content.

The basic idea of CVI-classifier is that a concept classifier is used to map local image features to middle-level semantic information^48^, and the annotation of global image as high-level semantic information is determined by scene classifier which is trained based on the meaningful middle semantic features. Considering the lesion size is an important basis for classification, multi-scale semantic modeling^42^ is introduced to extract meaningful middle-level semantic features which can describe image patches with different sizes properly. The flowchart of CVI-classifier is shown in Fig. 6. First, the pre-processing step including background remove and image normalization is utilized to eliminate the influence of background and lighting on the classification results. Image background is removed manually, and image normalization is achieved by gray world algorithm^49^. In future, since our follow-up work is applied to mobile devices, we can use leg contours as mask to cut images when capturing leg images. This process can remove image background easily. Second, the CVI images are divided into patches by multi-scale grids. Third, for each divided scale, the concept classifier is used to map the region features of image patch to the middle-level semantic features. Next, the image representation based on the concept occurrence frequency is calculated on each scale, and the image representations of all scales are concatenated as the multi-scale semantic model for final image representation. At last, scene classifier based on these multi-scale semantic features^50^ is adopted to classify the CVI’s severity which includes mild, moderate and severe classes. In addition, in order to improve the classification performance, the concept classifier and scene classifier are trained using optimized features achieved by high-order relevance based feature selection approach^51^.

This study was approved by the ethics boards of Shanghai Sixth People’s Hospital affiliated to Shanghai Jiao Tong University,and all experiments and methods were performed in accordance with the relevant guidelines and regulations of Chinese Society of Microcirculation. In addition, informed consent for participation was obtained from all participants or their relatives.

### Semantic Modeling for CVI Image

**Figure 7:**
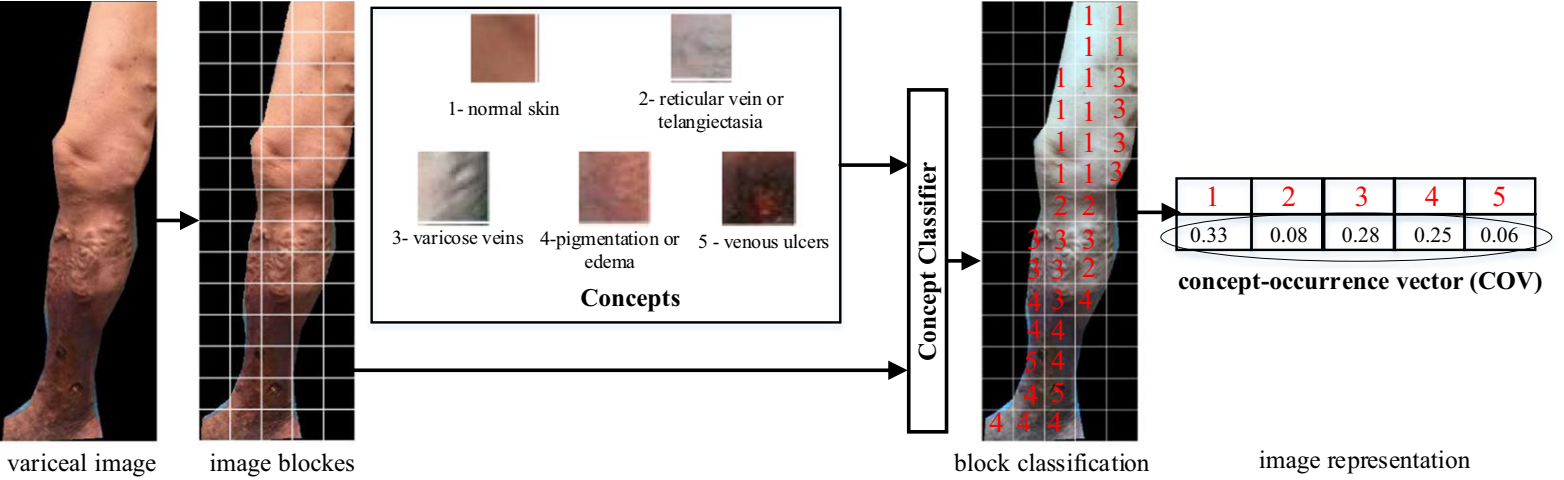
Semantic model for CVI image. 1 - normal skin, 2 - reticular vein or telangiectasia, 3 - varicose veins, 4 - pigmentation or edema and 5 - vein ulcers.

There may be more than one category of CVI in one lower limb image. In order to model this co-occurrence relationship, a semantic modeling approach based on local image patch is proposed^45^. This method contains three stages: concept selection, local region classification and global image representation. First, the concepts for semantic modeling are selected and notated in the image. The concepts that we use include normal skin, reticular vein or telangiectasia, varicose veins, pigmentation or edema, and venous ulcers, which are labeled from 1 to 5 respectively. Second, in order to be independent on the largely varying quality of an automatic segmentation, lower limb image is divided into regular grids. Then, the concept classifier based on support vector machine (SVM) is trained using the patch features and notated concepts. At last, the concept-occurrence vector (COV) which is a normalized histogram of the concept occurrences in the image is computed as image representation. The flowchart of the semantic modeling process is shown in Fig. 7.

**Figure 8:**
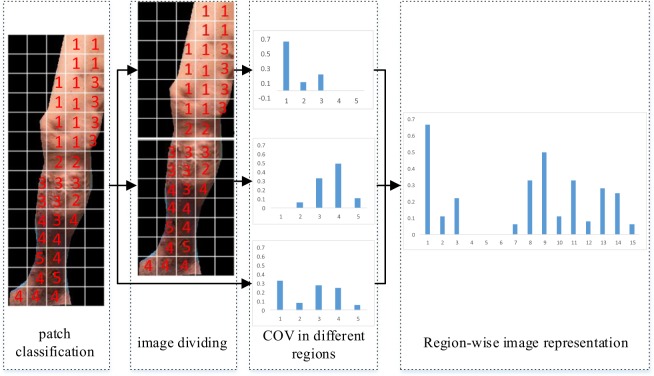
An example of computing the region-wise image representation. The image representation vector is denoted by the combination of the concept occurrence frequency calculated in upper, lower, and the whole image part respectively.

Medicals have found that 1) edema, skin changes or venous ulcers usually occur in ankle region, but may extend to leg and foot, 2) Spider veins often are found on thighs, 3) Varicose veins can be found on foot, the calf, the thigh or on the entire leg^52^. That’s to say, except the normal skin and varicose veins which may be found on the entire leg, other concepts can be divided into lower and upper groups according to the occurrence location. Therefore, in order to improve the discrimination, we divide the images in two and introduce region-wise image representation to reflect this phenomenon. An example for computing region-wise image representation is shown in Fig. 8. In this example, firstly, the CVI image is divided into upper and lower parts from the middle of image, which means that these two parts have same patches. Secondly, the concept distributions represented by concept occurrence frequency are calculated in upper and lower halves respectively. Thirdly, the COVs from upper half, lower half and whole region are concatenated as the fusion feature to represent original image. Since the concept distributions in upper half, lower half and whole region are various, the combined feature can reflect the image’s characteristics and is useful for classification.

Compared with Bag-of-Features methods^21^, our semantic model uses supervised learning algorithm for constructing the word dictionary. The advantages of semantic modeling are as follows: 1) by introducing the notated labels, the gap between the image understanding and the image representation is narrowed; 2) the dimension of the image representation vectors based on semantic modeling is lower than the one from the Bag-of-Features methods; 3) local information are collected in the COVs as the global representation.

### Image Representation by Multi-Scale Semantic Modeling

Multiple lesions with different sizes and different CVI categories may appear simultaneously in a CVI image. To get a proper description of region features in the image, the image should be divided into grids with appropriate grid size, otherwise, the extracted region features of image patches cannot represent the concepts whose sizes are significant different. Therefore, in order to extract distinguishable and proper region features which can reflect the characteristics of various concepts, the multi-scale semantic modeling for lower limb image is proposed. We believe that this model will lead to more accurate concept classification that can improve the image representation.

**Figure 9:**
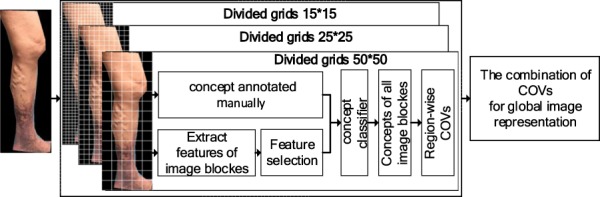
Image representation using multi-scale semantic modeling. The region-wise representation is computed at each divided scale, and the final image representation is denoted by the combination of region-wise representation at all divided scales.

The basic idea of multi-scale semantic modeling for image representation is to concatenate the region-wise COVs at each divided scale. As shown in Fig. 9, this approach includes four steps: image divided by multi-scale grids, image patch features extracted and concept annotated manually, feature selection and concept classifier training, and the region-wise COVs computing and their combination for global image representation. In the first step, considering the importance of the proper size of the image patch for classifying specific CVI’s category, multiple grids with several sizes are used to divide the lower limb image. Based on the lesion size of different categories in CVI, 15 × 15, 25 × 25, and 50 × 50 grids are chosen for dividing.

In the second step, various types of region features are extracted to improve the representation of image patches. The kinds of region features include LBP (Local Binary Patterns)^53^, EHD (edge histogram descriptor)^54^, GLCM (gray-level co-occurrence matrix)^55^, the variance and mean of dense SIFT^56^ in the image block, the mean value of gradient^57^, the variance, median, and mean value of each channel in RGB and YCbCr color spaces^58^, and the contextual features of neighbor blocks^59^. In addition, due to the significant correlation between the lower limb CVI’s category and its position, the patch index is used as the patch feature. Compared with automatic feature extraction, the training sample concept is annotated manually. This annotated step consists of selecting concepts and professional annotation. The concepts of these training samples are defined in the subsection *Semantic Modeling for CVI Image*. In order to ensure the accuracy of labeling patch samples, these multi-scale image patches are labeled by three medical professionals of vascular surgery of Shanghai Sixth People’s Hospital affiliated to Shanghai Jiao Tong University. If the patch labels from three doctors are inconsistent, we adopt voting to make decisions. If two doctors’ opinions are the same, patch label is annotated based on their suggestions. For one patch, if the results of the three doctors are different from each other, we assign the label of its nearest patch.

In the third step, the concept classifier is trained to classify the patches of new samples. Considering the noisy features which reduce the relevance between the category and the features, and the redundant features which limit the generalization of the classifier, the optimized subsets of the region features are selected using a high-order relevance-based feature selection approach to improve prediction accuracy. Then, a multi-class SVM^60^ is trained to form the concept classifier.

In the final step, the global image representation is computed. Because of the correlation between CVI’s severity and its position, region-wise VOC is introduced for global image representation. The final image representation based on multiple scale semantic model is the combination of region-wise image representation at each divided scale. The combined image representation contains meaningful semantic information which is provided by the supervised labels from professional doctors. Moreover, these combined features involve local, global, and multi-scale information which are introduced by image patches, the concept-occurrence frequency, and the multiple divided scales. Therefore, these combined features are more efficient and representative.

### Feature Selection and Scene Classifier

At each divided scale, the grid size does not match all CVI’s concepts. Therefore, some features in the combined features may be efficient, while others may be redundant. Moreover, due to the classification errors in classifying the image patches using the concept classifier automatically, some features among these combined features may contain noises, and cannot provided sufficient discriminative information. In order to remove these redundant and noisy features which reduce the classifier’s accuracy and generalization ability, we propose a high-order dependency based feature selection approach, which can take into account the relevancy and redundancy among several variables and the relevancy between the variables and the specific classification task.

**Figure 10:**
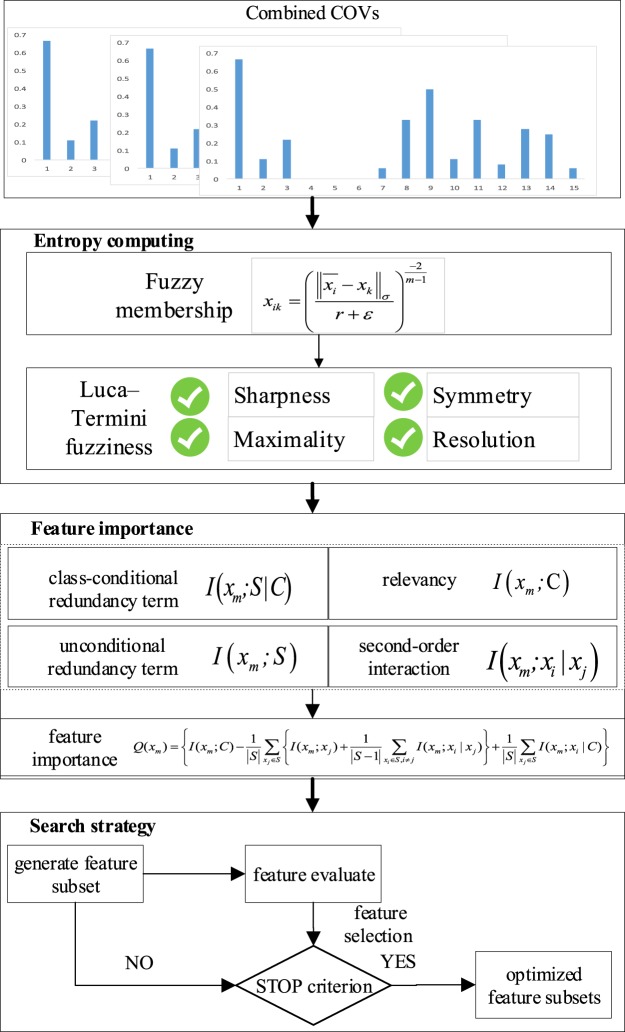
The flowchart of the feature selection approach. The feature importance is evaluated using the combination of the class-conditional redundancy term, unconditional redundancy term, relevancy term and second-order interaction.

This feature selection method (shown in Fig. 10) has three stages: entropy computing, feature importance evaluating and search strategy. First, joint entropy, conditional entropy and marginal entropy are computed based on joint, conditional, and marginal probabilities respectively. Since estimating these probabilities is very difficult, the fuzzy membership as alternative and the Luca–Termini entropy are used to calculate the joint fuzzy entropy, conditional fuzzy entropy and marginal fuzzy entropy^61^. Second, the feature significance is measured by the novel criterion based on the high-order dependencies which consider class-conditional redundancy term, unconditional redundancy term, relevancy term and second-order interaction^51^. Third, the forward search strategy is adopted to find an optimized feature subset^62^.

Then, the scene classifier is trained to determine the severity of CVI. Taking into account doctor’s treatments for CVI of different severity (for example, a mild patient does not need to handle or wear elastic stockings, a moderate patient needs simple surgery, and a severe patient needs complicated surgery), the mild, moderate and severe situations are used to label the global image. In this paper, mild class means that non-symptom or reticular vein or telangiectasia, moderate class denotes varicose vein, and severe class contains edema, eczema and venous ulcer. After obtaining an optimized feature subset for more efficient image representation, a multi-class SVM^60^ is trained for scene classification.

## Data Availability

The image dataset and relevance labels can be downloaded from http://isyslab.info/CVI/CVI-img-datasets.zip and https://github.com/shiqiangdqq/CVI-classifier/tree/master/CVI-imgdatasets.
